# Nonspecific lower back pain in overhead athletes is multifactorial and is not solely determined by the type and intensity of the sport

**DOI:** 10.17159/2078-516X/2026/v38i1a24875

**Published:** 2026-03-15

**Authors:** J Finsterer

**Affiliations:** Neurology Department, Neurology and Neurophysiology Centre, Vienna, Austria

We read with interest the article by Moatshe et al. on a cross-sectional study of the prevalence and risk factors of nonspecific low back pain (NSLBP) in amateur athletes who regularly participate in sports involving repetitive overhead movements.^[1]^ Among the 52 patients examined, the prevalence of NSLBP was 25%; 48% had scapular dyskinesia; and 40% had limited flexibility of the latissimus dorsi.^[1]^

The study is interesting, but some points should be discussed. The first point is that the origin of NSLBP is not limited to the lumbar spine but is multifocal. NSLBP can originate not only from muscles, joint capsules, tendons, ligaments, and cartilage, but also from other tissues and structures equipped with nociceptors. These include the kidneys, ureters, arteries, bladder, prostate, uterus, ovaries, and adnexa.^[2]^ It is therefore important to include all these tissues in the differential diagnosis of NSLBP. It is equally important to include dehydration, chronic infections, endometriosis, electrolyte imbalances, endocrine disorders, chronic renal failure, and stones in the differential diagnosis.

The second point is that NSLBP can be triggered not only by sporting activities, but also by everyday activities. It is therefore important to know the type and intensity of the work performed by the test subjects and what other activities they engage in besides sports when they are not working. A person building a house or renovating their apartment is more likely to develop NSLBP than someone whose hobby is playing chess. A person with a sedentary job is less likely to develop NSLBP than a person who works as a construction worker, blacksmith, or carpenter.

The third point is that NSLBP may also be due to side effects of the current medication or diet that a subject takes regularly. Therefore, it is important to include diet and current medication in the analysis. How many of the included participants regularly or occasionally took analgesics, sedatives, hypnotics, antidepressants, or antiepileptics, and how many of them were on a specific diet (e.g., low-carbohydrate, anaplerotic, ketogenic, low-fat, vegetarian, vegan, etc.)? How many of the participants used illegal drugs?

The fourth point is that the prevalence of NSLBP may also depend on the type of overhead sports. Since slopestyle snowboarders, climbers, and fistball players may be exposed to different stresses compared to volleyball, basketball, netball, tennis players, and swimmers, it is conceivable that the choice of overhead sport for analysis determines the prevalence of NSLBP.

The fifth point is that the quality and quantity of sleep can greatly influence the frequency of NSLBP.^[3]^ Therefore, the assessment of sleep performance should be included in the analysis. It is also important that the extent of acute and chronic stress and depression be included in the analysis, as not only physical overload but also psychological overload can influence the frequency and intensity of NSLBP.^[4]^ In this sense, it is also recommended to include personality type, the availability of coping strategies for life’s injustices, and social behaviour in the analysis.

Overall, assessing the frequency of NSLBP in amateur athletes who perform overhead movements requires the inclusion of all factors that cause, trigger, modify, or mimic NSLBP in these athletes.

## References

[1] Moatshe TP, Dawood M (2025). Prevalence and risk factors of nonspecific low back pain among amateur overhead athletes in Gauteng Province. S Afr J Sports Med.

[2] Farley T, Stokke J, Goyal K, DeMicco R (2024). Chronic Low Back Pain: History, Symptoms, Pain Mechanisms, and Treatment. Life (Basel).

[3] Skarpsno ES, Nilsen TIL, Mork PJ (2021). The effect of long-term poor sleep quality on risk of back-related disability and the modifying role of physical activity. Sci Rep.

[4] Choi S, Nah S, Jang HD, Moon JE, Han S (2021). Association between chronic low back pain and degree of stress: a nationwide cross-sectional study. Sci Rep.

